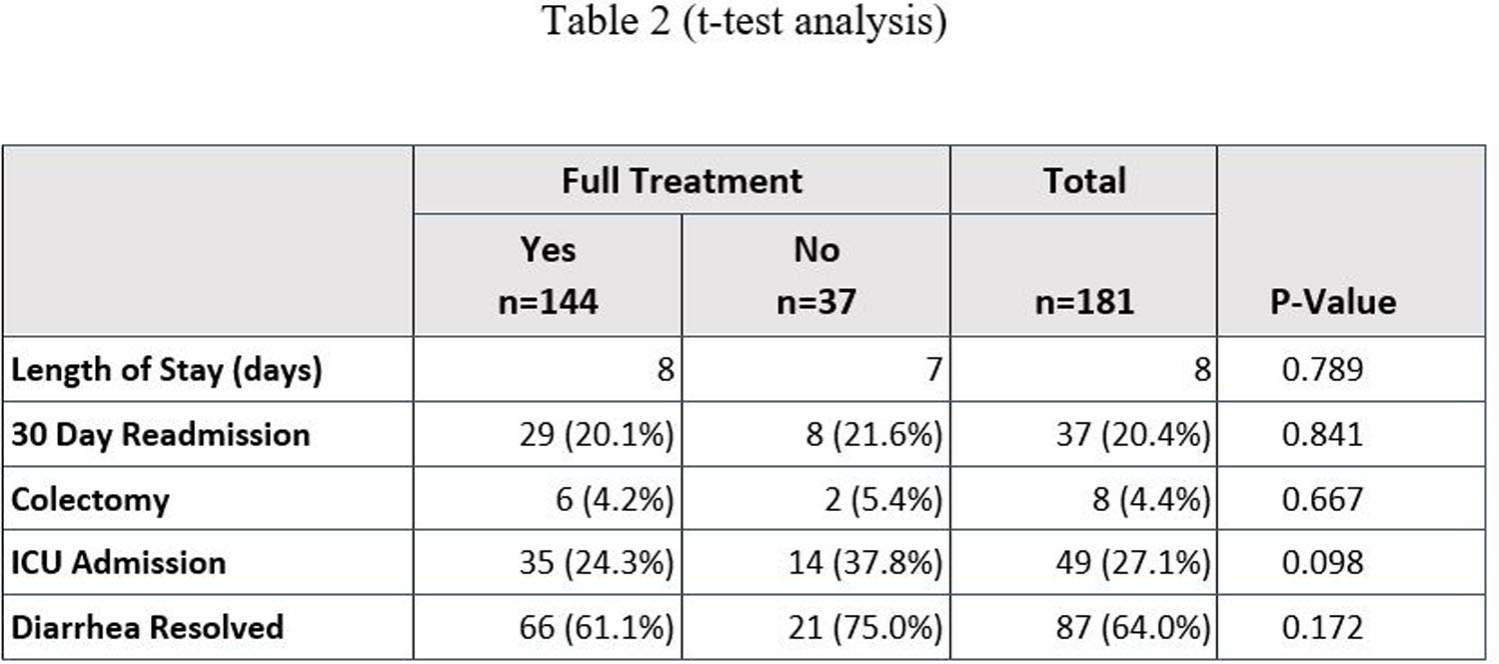# Assessing Clinical Outcomes for C. diff Polymerase Chain Reaction (PCR)-positive, Enzyme Immunoassay (EIA)-negative Patients

**DOI:** 10.1017/ash.2024.183

**Published:** 2024-09-16

**Authors:** Philip Jurasinski, Xiangni Wu, Ifrah Fatima, Greeshma Erasani, Barbara Dyer, Nicholas Bennett, Sarah Boyd

**Affiliations:** University of Missouri-Kansas City; St Lukes Health System of Kansas City

## Abstract

**Background:** In August 2021, Saint Luke’s Health System (SLHS) transitioned Clostridioides difficile (C. diff.) testing from polymerase chain reaction (PCR)-only to two-step enzyme immunoassay (EIA) reflex following PCR+ for suspected C. diff. infection. Uncertainty in patient management may arise when PCR and EIA testing differ. Previous studies suggested that disease severity varies when a patient’s results demonstrate PCR+ and EIA- due to possible colonization. Clinicians may not treat if diarrhea self-resolves, patients remain stable, or alternate causes of diarrhea exist. We compared clinical outcomes of patients who received treatment to those who did not. **Methods:** This was a retrospective cross-sectional study from August 2021-August 2023 in a multi-site, integrated health system, comparing 181 inpatients with PCR+/EIA- C. diff. test results stratified by no treatment (0-48 hours of C. diff. targeted treatment), partial treatment (2-9 days), or full treatment (10+ days). The primary outcome was length of stay. Secondary outcomes were readmission rates, need for colectomy, intensive care unit (ICU) admission, and diarrhea resolution on day of discharge. **Results:** Of the 181 patients, 144 received full treatment, 17 had partial, and 20 had no treatment. Baseline characteristics were similar between groups. No significant difference was found for length of stay or any secondary outcomes (Table 1). Table 2 provides a subgroup of patients who received no treatment vs those receiving partial or full treatment. **Conclusion:** In this study, treatment exposure did not affect clinical outcomes for patients with PCR+/EIA- results, though sample sizes may limit generalizability. Further research is warranted regarding the clinical approach to PCR+/EIA-

**Figure:**
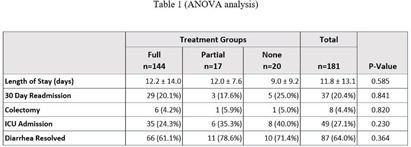

**Figure:**